# Platelet adhesion and aggregate formation controlled by immobilised and soluble VWF

**DOI:** 10.1186/s12860-020-00309-7

**Published:** 2020-09-11

**Authors:** Matthias F. Schneider, Mohammad A. Fallah, Christian Mess, Tobias Obser, Reinhard Schneppenheim, Alfredo Alexander-Katz, Stefan W. Schneider, Volker Huck

**Affiliations:** 1 Department of Physics, Medical and Biological Physics, Technical University Dortmund, Emil-Figge-Str. 50, 44227 Dortmund, Germany; 2 Department of Chemistry, University of Konstanz, Universitätsstr. 10, 78457 Constance, Germany; 3 University Medical Centre Hamburg-Eppendorf, Centre for Internal Medicine, Martinistr. 52, 20246 Hamburg, Germany; 4 Department of Paediatric Haematology and Oncology, University Medical Centre Hamburg-Eppendorf, Martinistr. 52, 20246 Hamburg, Germany; 5 Department of Materials Science and Engineerin, Massachusetts Institute of Technology, 400 Technology Sq. (NE46-605), Cambridge, MA 02139 USA; 6 Heidelberg University, Medical Faculty Mannheim, Experimental Dermatology, Theodor-Kutzer-Ufer 1-3, 68167 Mannheim, Germany

**Keywords:** Von Willebrand factor, Platelet adhesion, Shear activation, Primary haemostasis

## Abstract

**Background:**

It has been demonstrated that von Willebrand factor (VWF) mediated platelet-endothelium and platelet-platelet interactions are shear dependent. The VWF’s mobility under dynamic conditions (e.g. flow) is pivotal to platelet adhesion and VWF-mediated aggregate formation in the cascade of VWF-platelet interactions in haemostasis.

**Results:**

Combining microfluidic tools with fluorescence and reflection interference contrast microscopy (RICM), here we show, that specific deletions in the A-domains of the biopolymer VWF affect both, adhesion and aggregation properties independently. Intuitively, the deletion of the A1-domain led to a significant decrease in both adhesion and aggregate formation of platelets. Nevertheless, the deletion of the A2-domain revealed a completely different picture, with a significant increase in formation of rolling aggregates (gain of function). We predict that the A2-domain effectively ‘masks’ the potential between the platelet glycoprotein (GP) Ib and the VWF A1-domain. Furthermore, the deletion of the A3-domain led to no significant variation in either of the two functional characteristics.

**Conclusions:**

These data demonstrate that the macroscopic functional properties i.e. adhesion and aggregate formation cannot simply be assigned to the properties of one particular domain, but have to be explained by cooperative phenomena. The absence or presence of molecular entities likewise affects the properties (thermodynamic phenomenology) of its neighbours, therefore altering the macromolecular function.

## Background

The shear dependent role of von Willebrand factor (VWF) during primary haemostasis is very well established and investigated [1–10]. Understanding the VWF function is consisted of the physical aspects of hydrodynamics and structural conformations, and physiological aspects ranging from the underlying molecular biology to its functional characteristics and clinical impact.

Clinically, both qualitative (Type II) and quantitative (Type I and III) VWF variants are classified in the framework of von Willebrand disease (VWD) [11–13]. VWD as a hereditary disease is a common bleeding disorder caused by mutations of VWF resulting in deficiency or dysfunction of this biopolymer. In addition, the acquired von Willebrand factor syndrome (AVWS), reviewed by Tiede and coworkers, subsumes diverse non-inherited qualitative, structural or functional VWF disorders resulting in an enhanced risk of bleeding [14, 15]. The hereditary VWD certainly affects both the immobilised and the soluble VWF fractions, whereas AVWS leads to an impact only on the mobile fraction. Although the first step of haemostasis depends on the presence of an intact immobilised binding partner for platelets in the subendothelial vessel wall [16–18], bleeding episodes of patients suffering from AVWS illustrate that the mobile VWF fraction is a prerequisite for an effective VWF triggered blood clotting. Independent of the different pathomechanisms and clinical manifestations, the similar symptomatic therapeutic regimens of these syndromes underline the central role of VWF’s functional characteristics.

Bridging the gap between VWF’s clinical impact and its physical mechanistic background, former studies on single VWF molecules under flow [9, 19] as well as on its collective behaviour [20–23] have further elucidated the role of VWF while in motion. The impact of specific VWF domains on its main binding partners in the human vasculature, such as VWF itself [24–26], collagen [27, 28] and in particular platelets via shear-dependent VWF A1-domain to glycoprotein (GP) Ib interactions [24, 29, 30], have been investigated in detail for both realising basal mechanistical insights and as potential therapeutic targets in the wide field of haemostaseology.

**Fig. 1:**
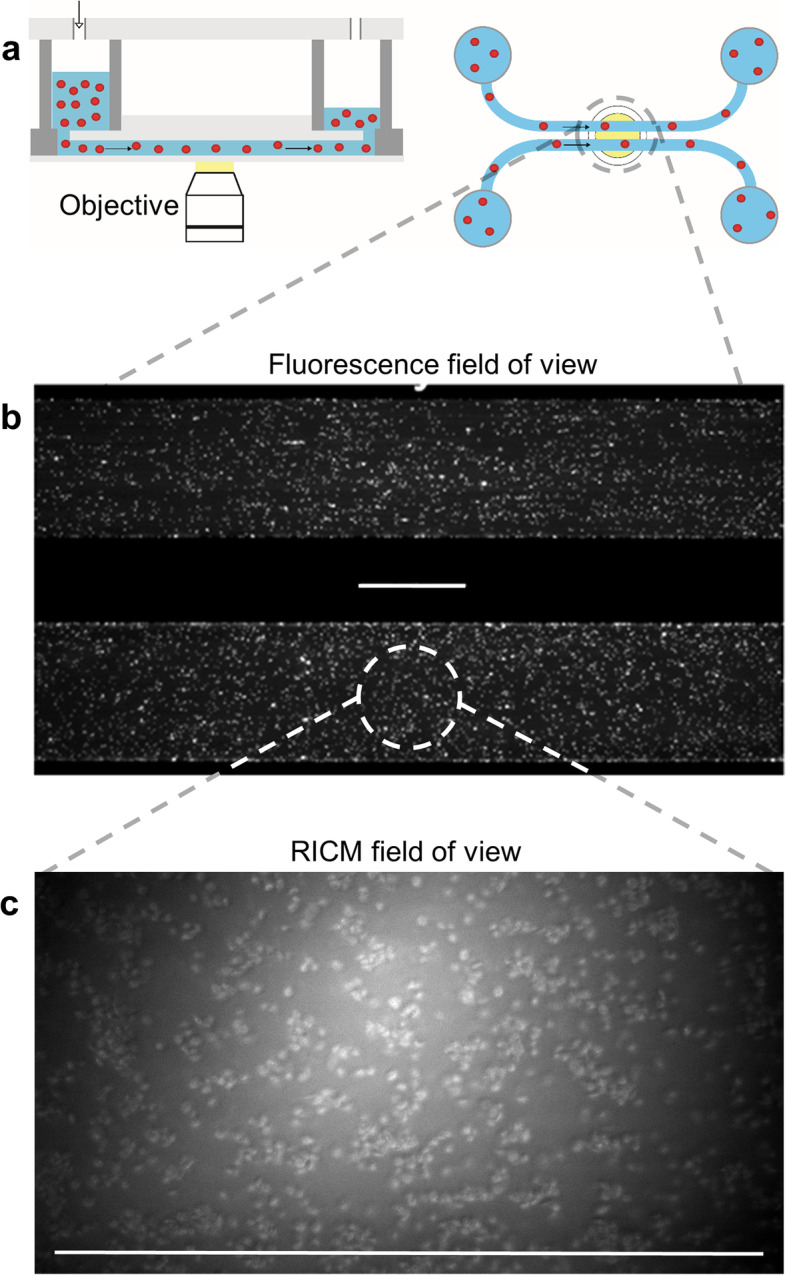
Combination of microfluidics and microscopy. **a** Left: A schematic sketch of the pneumatically driven BioFlux flow chamber mounted on the top of the Zeiss Axio Observer microscope, which is operated in fluorescence mode, or reflection interference contrast mode (polarised monochromatic light). Right: Flow is driven by air pressure from the reservoir into the straight duct. Two channels can be screened simultaneously in the field of view (dashed circle). **b** Fluorescence microscopic image of washed platelets interacting with the footprint of two independent parallel channels under flow conditions. Both channels are coated with wt VWF. **c** RICM image of platelet adhesion in whole blood under flow conditions. The channel is coated with wt VWF. Scale bars correspond to 200 μm

In this study, we combined a microfluidic setup with fluorescence- and reflection interference contrast microscopy (RICM) (see Fig. 1) to compare the impact of various deletions on VWF’s A-domain on the physiological function of both immobilised and soluble VWF. Large VWF-platelet-aggregates of several 100 μm in size, formed *reversibly* above a critical shear threshold, begin to roll on the surface of a microfluidic channel under whole blood conditions [31, 32]. These data demonstrate once more the importance to approach blood clotting as a dynamic process, in which in general important conformations - or better states - of all contributing constituents are a function of all physical (here shear flow) and biochemical (e.g. pH, multimer size) conditions. Systematically deleting the A1-, A2- or A3-domain of the surface-bound (immobilised) and mobile (soluble) VWF fraction, while studying platelet adhesion and reversible VWF-platelet aggregate formation over a wide range of shear rates, revealed the non-trivial interrelation between these domains, and showed that similar adhesion characteristics can be accompanied by a significant shift in aggregation tendency depending on the specific mutation.

## Results

### Platelet adhesion on immobilised VWF

**Fig. 2:**
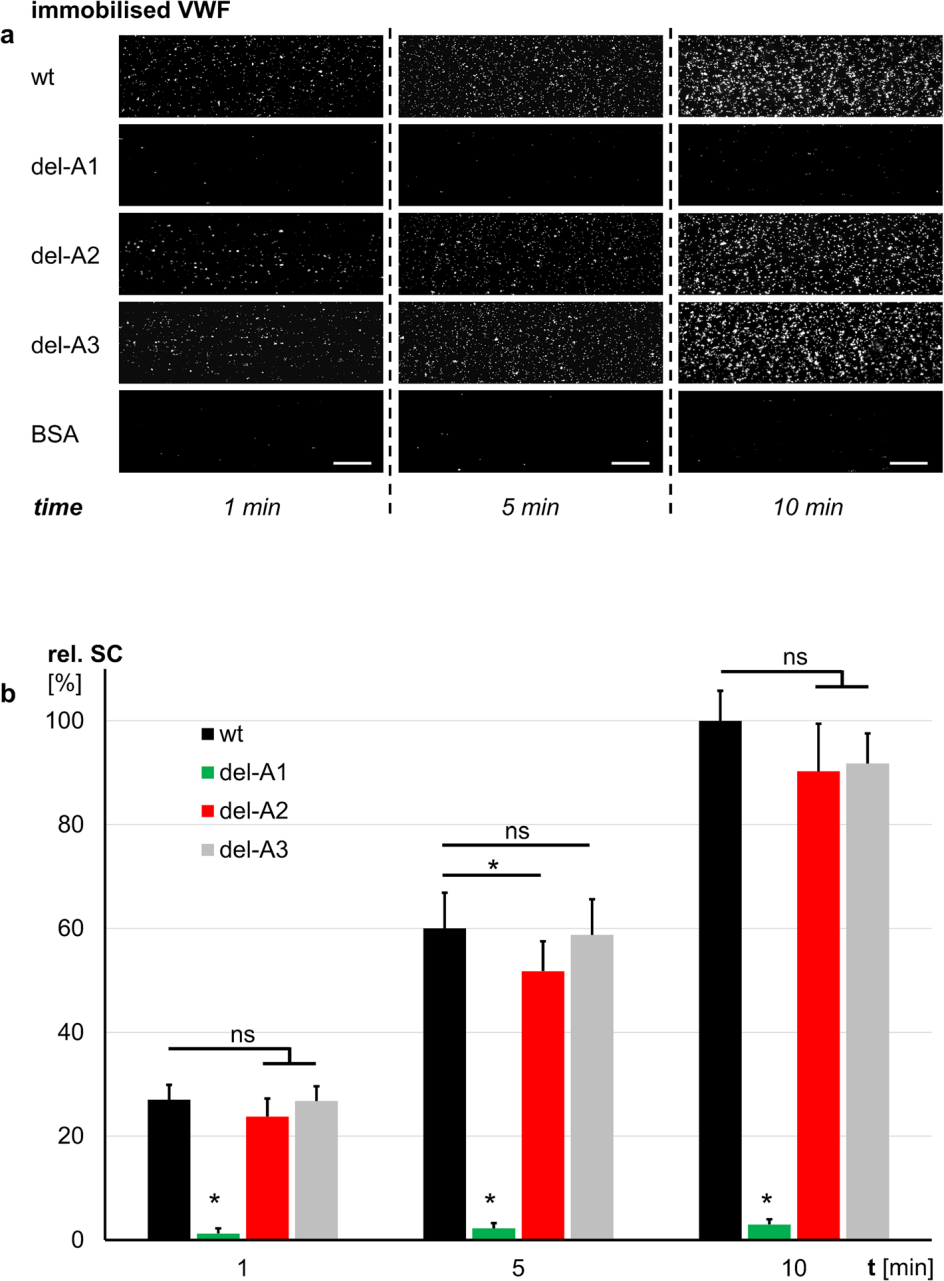
Platelet adhesion on immobilised VWF. **a** After biofunctionalisation of the microfluidic channels with the three different VWF A-domain deletion variants or wt VWF, the adhesion characteristics of platelets are measured upon constant shear rate application of 500 s^− 1^ at indicated points in time. Representative images out of four independent experiments for each group are depicted. As expected, coating with wt VWF leads to an increasing platelet adhesion over time. Del-A1 VWF coating fails to bind platelets. The slight change of platelet adhesion to del-A2 remained negligible compared to the wt VWF. Platelet adhesion to del-A3 coating is similar to wildtype. Biofunctionalisation with BSA serves as negative control. Scale bars correspond to 100 μm. **b** The relative platelet surface coverage (SC) to wt VWF on indicated biofunctionalisation is plotted against time. Wt VWF (black column), del-A3 (grey column) and del-A2 (red column) show similar platelet adhesion characteristics over time. Del-A1 (green column) nearly fails to bind platelets comparable to BSA serving as negative control (data not shown). n > = 4 for each experimental group, * indicates *P* < 0.05

Focusing on the shear conditions present in the human microvasculature [33] we applied shear rates between 500 s^− 1^ and 10,000 s^− 1^. Figure 2 plots the adhesion of platelets under low shear conditions as a function of time for the three different VWF A-domain deletion variants (del-A1, del-A2, del-A3) in the absence of soluble VWF. Whereas biofunctionalisation with wt VWF led to a time-dependent increase in platelet adhesion, coating with del-A1 VWF mutant failed to bind platelets, comparable to biofunctionalisation with bovine serum albumin (BSA) serving as negative control. Albeit the complete deletion of entire subdomains, there was no significant difference between del-A3 and wt VWF. The slight change of platelet adhesion to del-A2 remained negligible compared to the wildtype glycoprotein. These platelet adhesion characteristics were quantified by the surface coverage (SC) of platelets at the footprint of the biofunctionalised channels relative to wt VWF as previously published [34] (see Fig. 2b).

**Fig. 3:**
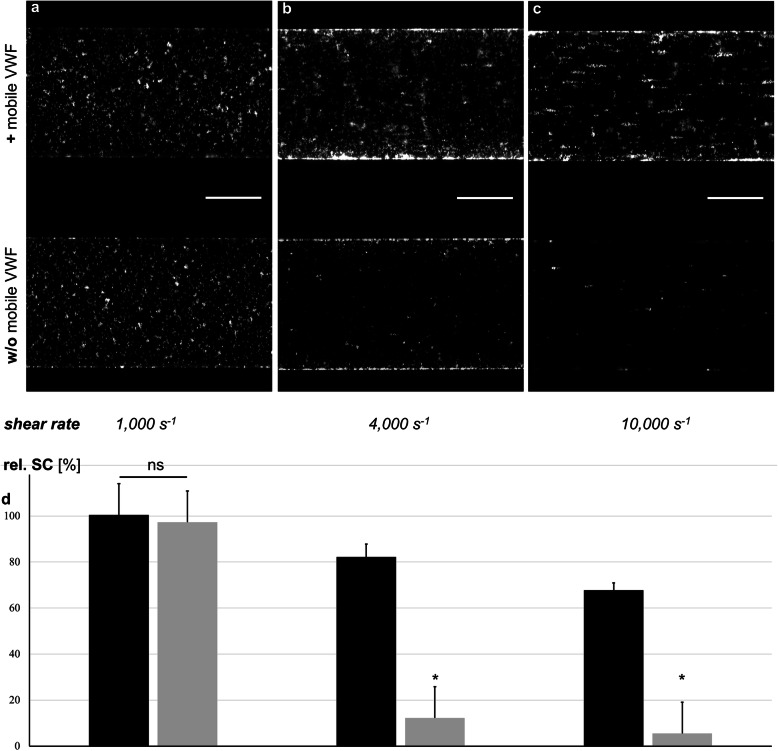
Role of soluble VWF in platelet adhesion from low to high shear conditions. Wt VWF biofunctionalised channels are perfused with washed platelets with (+, upper channels) or without (w/o, lower channels) addition of mobile wt VWF at distinct shear rates in the range of 1000 s^− 1^ to 10,000 s^− 1^. No differences are found between these experimental groups regarding the platelet SC at the low shear rates in the stated range (**a**). In contrast, at high shear rates platelets only adhere to the immobilised VWF in the presence of mobile VWF (**b**, **c**). Representative images out of five independent experiments for each group. Scale bars correspond to 200 μm. (**d**) The relative platelet surface coverage (SC) to wt VWF at a shear rate of 500 s^− 1^ is plotted against distinct shear rates as indicated in presence (black columns, +) or absence of mobile wt VWF (grey columns, w/o). *n* = 5 for each experimental group, * indicates P < 0.05

Keeping the immobilised VWF fraction unchanged, we next addressed the role of soluble VWF in the process of platelet adhesion. Therefore, we performed flow experiments on channels biofunctionalised with wt VWF, with or without addition of mobile wt VWF at distinct shear rates in the range of 1000 s^− 1^ to 10,000 s^− 1^. As shown in Fig. 3, there were no qualitative differences between these experimental groups regarding the platelet SC at low shear rates (Fig. 3a). But interestingly, at higher shear rates platelets could only adhere to the immobilised VWF in the presence of soluble VWF (Fig. 3b and c), quantified in Fig. 3d. These data illustrate that the mobile VWF fraction is a prerequisite for platelet adhesion under high-shear conditions.

### Collective behaviour: mobile VWF-induced aggregate formation

**Fig. 4:**
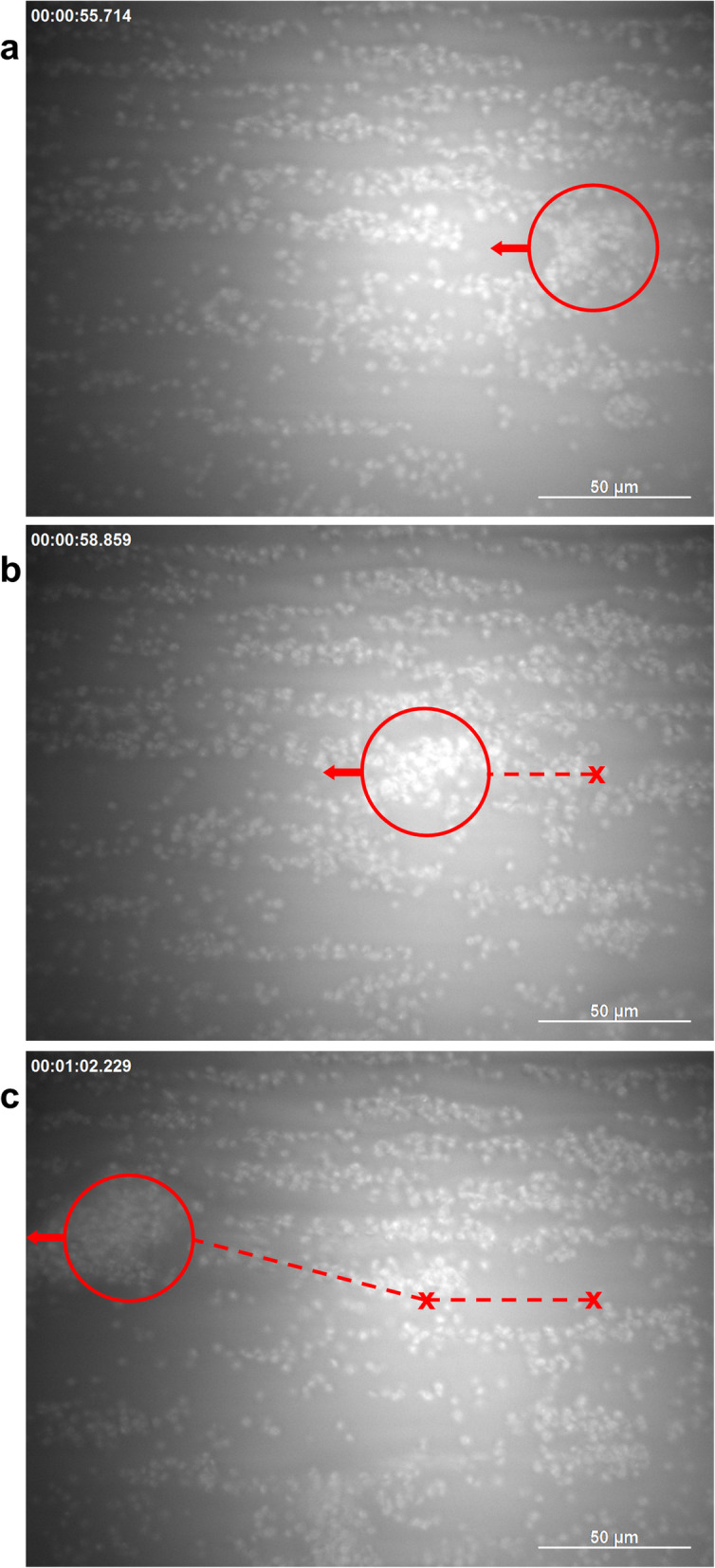
and Additional file 1 The process of aggregate formation on a wt VWF coated channel footprint. An RICM time-lapse live-cell movie (Additional file 1) is recorded at the footprint of a wt VWF biofunctionalised channel perfused with whole blood supplemented with wt VWF. The bright contrasted objects in the focal plane are platelets interacting with VWF at the channel surface. Note that only events in the direct adjacency to the focal plane are visible in RICM. Upon continuously increasing the shear rate within the first 35 s, the pattern of single rolling platelets switches to platelet decorated string-like structures (~ t = 20 s) which progressively stretch in flow. After some of these strings detach and first aggregates emerge (at the critical shear rate of 4000 s^− 1^), the flow was stopped leading to an immediate disassembly of strings and aggregates. From t = 37 s, we restart the flow with a constant shear rate of 4000 s^− 1^, resulting in re-formation of aggregates rolling along the surface. Still images at t = 56 s (**a**), t = 59 s (**b**) and t = 62 s (**c**) illustrate the tracking of one exemplarily chosen rolling VWF-platelet aggregate (red circles). Time frame in seconds as indicated, scale bar corresponds to 50 μm

Recently we could show that under whole blood conditions large VWF-platelet aggregates reversibly formed above a critical shear threshold rolling alongside the surface of a microfluidic channel [31, 32]. For the process of rolling aggregate formation see Fig. 4 and the RICM time-lapse live-cell movie (Additional file 1).

**Fig. 5:**
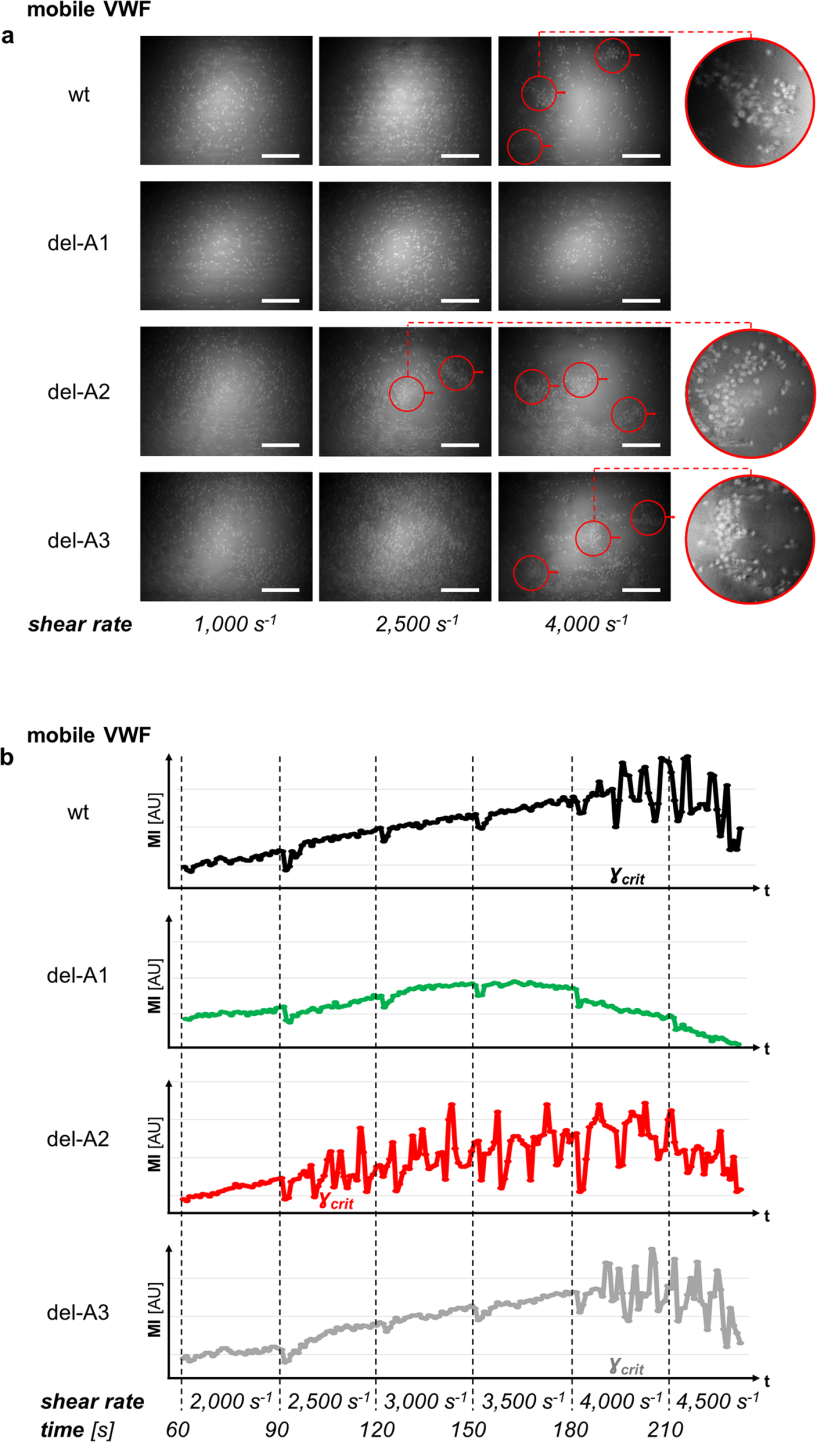
Critical shear rate of rolling aggregate formation depends on mobile VWF fraction. After biofunctionalisation with wt VWF, channels are perfused with whole blood supplemented with indicated mobile VWF variant and observed using RICM. The critical shear rate for VWF-platelet aggregate formation is analysed by consecutively increasing the shear from 1000 s^− 1^ to 5000 s^− 1^ in steps of 500 s^− 1^ (**a**) Whereas the VWF A1-domain deletion completely fails to induce rolling aggregates, del-A2 shows a gain of function by decreasing the critical shear compared to wt VWF; del-A3 does not influence the critical shear rate. Red-arrowed circles illustrate aggregates rolling on the surface in the marked direction. In the right column, magnifications of rolling aggregate regions at the critical shear rate are depicted if available. Scale bars correspond to 50 μm. (**b**) The RICM signal intensities (MI [AU]) for wt VWF (black line), del-A1 (green line), del-A2 (red line) and del-A3 (grey line) of the VWF-platelet aggregates are plotted against the time, representing distinct shear rates as indicated. Maximum fluctuations in the intensity plots correspond to the critical shear rate *ɣ*_*crit*_. The analysed *ɣ*_*crit*_ for del-A2 VWF is about 40% less than for wt VWF. Note that the detection of fluctuations as transition point representing *ɣ*_*crit*_ has two consequences: 1. The plotted intensities have to be representative tracings out of four independent experiments for each group as a graphical overlay would naturally mask this transition point. 2. With the discrete step width of 500 s^− 1^ the single experiments of each experimental group showed exactly the same depicted transition point. Therefore, *ɣ*_*crit*_ for del-A2 is significantly lower than *ɣ*_*crit*_ for wt VWF and del-A3 VWF. While the certainty is predominantly determined by the step width, we avoid indicating a *P* value

In Fig. 5a, a sequence of images along increasing shear rates is shown, where in addition to surface-bound immobilised VWF, VWF was also present in solution (mobile). Deletions of an individual domain showed distinct consequences on the aggregate formation: While A1-deletion mutant completely failed to induce VWF-platelet aggregates on an intact wt VWF biofunctionalised surface, the A2- and A3-deletion mutants are capable of aggregate formation. Compared to addition of wt VWF, del-A2 formed aggregates even at lower shear rates, clearly indicating a gain of function. A meaningful quantitative measure of aggregate formation has been shown to be the critical shear rate (*ɣ*_*crit*_), which in principle determines a nucleation point [20]. To quantify *ɣ*_*crit*_ next to the optical identification of first rolling aggregates (see Fig. 5a, red circles), we plotted the RICM signal intensities of aggregated platelets against the time, here representing distinct shear rates (Fig. 5b). Maximum fluctuations in the intensity plots correspond to *ɣ*_*crit*_.

## Discussion

In this study, it was clearly shown that, in order to determine the influence of VWF in the first step of haemostasis, solely the quantification of the platelet adhesion – using in vitro standard procedures – is not sufficient. Only the comprehension of the biophysical component of the fluid dynamics on the one hand, the integration of both collective and single molecule phenomena on the other hand, and finally the distinction between immobilised and soluble VWF opens up the possibility for a targeted investigation of the mechanistic background.

Platelet adhesion on immobilised VWF is a time dependent process. To study the initial step of platelet adhesion due to interactions of the VWF A1-domain and the platelet glycoprotein (GP) Ib, platelet activation was inhibited as previously described [35]. Thus, platelet-derived VWF was excluded as a further source of the mobile VWF fraction. As expected, biofunctionalisation with the deletion mutation del-A1 succeeded in coating but completely failed to bind platelets. Regarding the platelet adhesion capability on immobilised VWF, the deletion mutations del-A2 and del-A3 did not show any significant variation compared to wt VWF. As bleeding events are known to cause pathological high-shear conditions in the area directly affected by the damaged vessel [36], we next focused on short-termed VWF-platelet interactions upon different shear flow regimens. High shear rates in human arterioles range around a few 100 s^− 1^ to 1000 s^− 1^ [33, 37, 38], and shear supported (human) platelet adhesion has been demonstrated to be effective already in the range of 500 s^− 1^ [9, 31, 39]. Under low- or intermediate-shear conditions, the impact of the mobile VWF fraction on platelet adhesion to VWF biofunctionalised surfaces was negligible. Nevertheless, in the entire absence of soluble VWF, platelet binding to the surface was significantly diminished upon high-shear application (see Fig. 3). In contrast, in the presence of soluble VWF rising shear led to an enhanced platelet adhesion. In accordance with former publications [40, 41], platelet decorated strings appear within seconds (see Electronic Supplementary Material 1), most likely due to a recruitment of stretched mobile VWF to the platelet surface. The further *fortune* of the formed reversible aggregates – assuming constant hypercritical shear conditions – will indeed be influenced by further VWF-VWF [25, 26, 42] and VWF-platelet interactions [23, 29, 30] as well as by a subsequent activation of the involved platelets. But focusing on the mechanistical background of the very initial step of the reversible rolling aggregate formation process, namely the physically-driven collective phenomena of VWF, these aspects should be discussed in future studies.

**Fig. 6:**
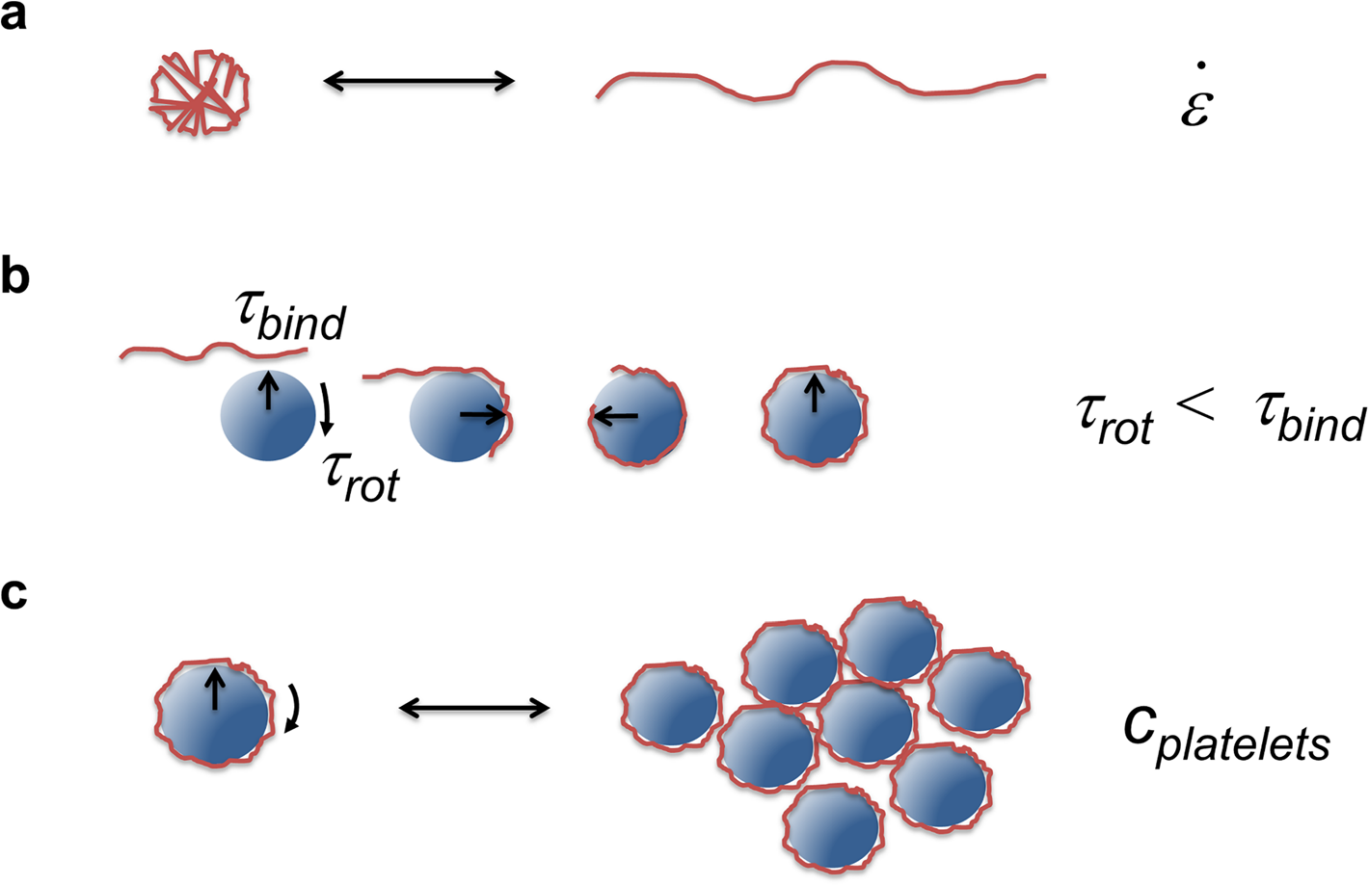
Proposed mechanism of aggregate formation. *VWF stretching.* Conformation of a single VWF molecule changes under high shear flow conditions from globular to elongated form. **b** *Cooperative association*. Stretched VWF binds to a rotating platelet. Only if the bond-lifetime *τ*_*bind*_ is sufficiently long (*τ*_*bind*_ > *τ*_*rot*_) compared to the period of rotation *τ*_*rot*_, VWF will begin to “wrap” around the platelet. This will allow forming multiple bounds, which stabilises the association against shear induced bond dissociation. **c** *Aggregate formation*. Partially VWF coated platelets will cross-interact with further platelets. If the platelet concentration c_platelets_ and hence collision rates are sufficient, this will initiate the reversible formation of aggregates

Although single platelet adhesion to the channel footprint and consecutive recruitment of soluble VWF are the likely prerequisites for aggregate formation, adhesion characteristics have no predictive power for the formation of rolling VWF-platelet aggregates. In a previous study, a truncated recombinant A1A2A3 tri-domain VWF was reported to have enhanced interactions with platelets compared to the full-length recombinant tri-domain VWF [22]. The truncated tri-domain VWF had a deletion of Gln^1238^-Glu^1260^ sequence of the flanking A1-domain. The deletion was reported to reduce the ‘masking’ effect of the VWF D’D3-domain on the GPIb interactions with VWF. In our work, the deletion of the A2-domain does not affect the platelet adhesion to immobilised VWF, nevertheless enhancing the formation of reversible VWF-platelet aggregates in the presence of a soluble VWF fraction. This indicates that the A2-domain also masks the reversible interactions of the platelets with the A1-domain of VWF. Our finding becomes more significant considering the fact that it has been previously reported that recombinant A2-domain polypeptides bind specifically to the VWF A1-domain [24]. This binding inhibits platelet adhesion by blocking the specific binding site of the A1-domain to the platelet GPIb. We could show for the selected deletion variants of the A-subdomains of VWF that a deletion of the A1-domain indeed led to a significant decrease in adhesion and a complete loss of aggregate formation. Nevertheless, while the impact of the A3-domain on adhesion and aggregate formation seemed to be marginal, a deletion of the A2-domain – although leading to a negligible change in platelet adhesion – induced a significant reduction in *ɣ*_*crit*_, i.e. an increase in the formation of VWF-platelet aggregates. Note that due to the absence of divalent cations during the whole course of microfluidic experiments, the activity of the VWF degradation enzyme ADAMTS-13, known to specifically interact with the VWF A2-domain, is inhibited to facilitate the concentration on the VWF interdomain affection. Although one could raise the hypothesis that adhesion and aggregate formation are regulated by specific binding sites on different parts along the molecule (e.g. the collagen binding site of the A3-domain) [43, 44], we here prefer to look at this molecule from a different more physical angle:

Our results strongly support a hypothetical scenario suggested earlier by Ruggeri et al. [6]: Single platelets tethered to immobilised VWF function as nucleation centres. Soluble VWF binds to the immobilised platelet, in particular under high shear flow, thereby representing a nucleation centre itself supporting the growth of the aggregate. The aggregate formation can be explained by a three-step process and the “matching” of two timescales. First, the elongational contribution of the shear field supports the elongated state of VWF (Fig. 6a). The shear field also introduces the first timescale *τ*_*rot*_ *≈ 1/* $\dot{\gamma}$ (gamma representing the shear rate), the period of one rotation of a platelet exposed to the field. The second timescale *τ*_*bind*_ arises from the binding kinetics and corresponds to the time the elongated VWF typically resides on the platelet. Of course, in reality, all these quantities are thermodynamic quantities and will vary with temperature, pH, ion concentrations etc. in other words they are represented by their appropriate diagrams of state [45]. Nevertheless, if *τ*_*rot*_ < *τ*_*bind*_ the VWF will reside long enough on the platelet to wrap around it (Fig. 6b). This will effectively coat the platelets due to the enlarged contact area. Via VWF-VWF interactions the platelets can now begin to form aggregates, given a high enough concentration of platelets (Fig. 6c).

We must note that a recent work explaining how VWF adheres to surfaces at high shear rates can also be used to explain why aggregate formation occurs only in the presence of soluble VWF [46]. In particular, at shear rates above 1000 s^− 1^, platelets are exposed to shear forces in the range above of 10 pN. This force is around the rupturing force of the GPIb-A1 bond [47]. Thus, in excess of this force platelets need a cooperative mechanism to bind. By forming aggregates, it is possible to overcome this limit since the binding to the substrate leads to a higher valence arising from the other platelets on the string. As long as the lifetime of the bonds is long enough, such aggregates will be able to bind and immobilise on the substrate.

The gain of function observed during aggregate formation for the del-A2 mutations reveals that the simple picture of altered VWF-platelet association (Fig. 2) leads to a false prediction of the aggregation behaviour. As shown in Fig. 5, the threshold shear rate at which aggregates form for wt VWF is of the order of 4000 s^− 1^; however, in the case of the deletion of the A2-domain it is 2500 s^− 1^. The ~ 40% change in this threshold shear rate can be accounted by an increase in the binding strength between the A1-domain and the platelet GPIb receptor. As has been previously speculated, we can confirm that the GPIb-A1 interaction is regulated by the presence of the A2-domain [21, 48]. It is believed that the interaction is masked by the A2-domain yielding an effective lifetime of the GPIb-A1 bond that displays a “catch-bond” behaviour [25, 29, 30]. In the absence of the A2-domain, the lifetime at lower shear forces of the GPIb-A1 bond was radically increased. By how much? Based on our previous work on aggregate formation [20], it is possible to measure such differences. In particular, in order to reduce the critical shear rate by *x*, the lifetime of the GPIb-A1 has to increase by the same factor *x*. By assuming that all prefactors remain the same, we find that the effective interaction strength between the GPIb and the A1-domain has to increase by ~ 1 kT, where k is the Boltzmann constant and T the temperature.

Such variation is small, yet has a dramatic impact on the clotting process. Thus, we predict that the A2-domain effectively “masks” the potential between the GPIb and the A1-domain by about 1 kT. While it would be hard to resolve an interaction energy of this magnitude, this change should not carry over into the high-shear regimen as the shear forces will already be enough to pull apart the domains and reduce the masking ability of the A2-domain.

## Conclusions

The results of our in vitro study contribute to a deeper understanding of the stepwise process of VWF activation and further clarify the impact of mobile VWF-initiated aggregate formation in the cascade of VWF-platelet interactions. Taken together, these findings call for a thermodynamic approach to understand VWF triggered blood clotting where the mechanical perspectives recently discovered [9, 49–53] are only a way towards an integrative perspective. In future, therefore it will be necessary to extract the phenomenology of VWF, here the changes of the protein’s state upon deletion mutations, by experiments, which are analysed thermodynamically and are the basis of a physical explanation of biological function as well as a gain to new insight on a microscopic scale.

## Methods

### Microfluidic channel system

The fluid dynamic conditions of the human microvasculature, was mimicked in a pneumatically driven microfluidic channel system (BioFlux, San Francisco, California, USA). The BioFlux system is a bench-top instrument, which allows long-term temperature-controlled flow cell assays. Its pressure interface connects a high precision electropneumatic pump to the well plates to initiate controlled flow rates with a nominal shear rate precision of 36 s^− 1^. The channel geometry is a straight rectangular duct with a width of 350 μm and a height of 75 μm. Biofunctionalisation succeeded by coating the channels with either wildtype von Willebrand factor (wt VWF) or a deletion mutant VWF in a concentration of 50 μg/ml each. A homogenous coating, representing the immobilised VWF at the extracellular matrix of a subendothelial vessel wall, was achieved after incubation over night at 37 °C in a moisture rich environment as previously published [20].

### Microscopic setup

The microfluidic channel system was mounted onto an inverted microscope (Zeiss Axio Observer Z.1, Zeiss AG, Oberkochen, Germany) operated in either fluorescence- or RICM-mode, respectively. RICM is especially beneficial for studying both dynamic and static biological phenomena taking place in vicinity of a transparent substrate, especially under whole blood experimental conditions. This microscopy mode is used to study the interference pattern of polarised incoming light being reflected at an object in order to reconstruct the height profile of the object at an interface as previously published [20]. Briefly, the interference of the object beam and the reference beams provides an image depicting sample’s height profile with high resolution. The reduction of scattered light succeeded in utilising the antiflex technique, in which the incident light passes a linear polariser. Therefore, the RICM technique assures detection of the platelet membrane coming in direct contact with the observed surface in a high spatial resolution [54]. This specific microscopy technique succeeded by an adapted utilisation of a 63fold antiflex EC Plan-neofluar objective, a reflector module Pol ACR P&C for HBO and a LED module Colibri illumination at 470 nm (all Zeiss AG, Jena, Germany). A sketch of this combined microfluidic/microscopic setting is shown in Fig. 1. Image acquisition was performed using a CCD camera (AxioCam MrM) and ZEN software (both Zeiss AG, Jena, Germany).

### Cell culture and expression of recombinant wildtype and deletion mutant VWF

For recombinant VWF expression, we used HEK293 cells (Deutsche Sammlung von Mikroorganismen und Zellkulturen, Braunschweig, Germany) as previously published [55]. In brief, cells were cultured in Dulbecco Modified Eagle Medium (DMEM, Invitrogen, Karlsruhe, Germany) supplemented with 10% foetal bovine serum and 1% penicillin/streptavidin at 37 °C and transfected with Lipofectamine 2000 (Invitrogen, Karlsruhe, Germany) and VWF-plasmid-constructs in vector pIRESneo2. Recombinant expression of VWF variants was performed as previously described [56]. In brief, HEK293 cells stably express wt VWF or indicated deletion mutants. Samples of the supernatant were taken after 72 h, centrifuged (5 min at 270 g, 4 °C) and concentrated with Amicon Ultrafree-15. The concentration of wt VWF, del-A1 (p.Glu1260_1480del) VWF, del-A2 (p.Asn1493_1673del) and del-A3 (p.Gly1672_1874del) VWF was determined by a polyclonal rabbit anti-human VWF:Ag-ELISA (DAKO, Hamburg, Germany). For a detailed description of its binding epitopes refer to Tan et al. [57].

### Preparation of the perfusion media

For preparation of the perfusion media, blood was smoothly collected from healthy volunteers after informed consent. We utilised sodium citrated monovettes with manual syringe stamps to avoid platelet preactivation and further inhibit the activity of inherent degradation enzymes (e.g. ADAMTS-13). This study, approved by the Ethics Committee II of the Heidelberg University (Mannheim, Germany), was conducted in conformity to the *Declaration of Helsinki* [58], to *The International Conference on Harmonisation of Technical Requirements for Registration of Pharmaceuticals for Human Use (ICH)* Guidelines and to the Convention for the Protection of Human Rights and Dignity of the Human Being with regard to the Application of Biology and Medicine: Convention on Human Rights and Biomedicine (Oviedo, 4 April 1997).

Platelets were isolated from blood samples, washed and fluorescently stained as previously published [35]. These platelets were resuspended in divalent cation free phosphate-buffered saline solution and used for perfusion in a concentration of 200,000 per μl supplemented with 45% haematocrit. Alternatively, the blood samples were natively perfused as citrate-anticoagulated whole blood supplemented with wt VWF or deletion mutant VWF as indicated.

### Experimental procedure

Under physiological conditions, VWF comes into effect in both the extracellular matrix of the subendothelial vessel wall (immobilised) and the circulating plasma (soluble) [4, 6–8]. Three sets of experiments were designed to discretely mimic the complex physiology of VWF mediated platelet adhesion and VWF-platelet aggregate formation in vitro:

1. In order to investigate the impact of deletions in the VWF A-domains on platelet adhesion, microfluidic channels were biofunctionalised with indicated deletion mutant VWF and perfused with washed platelets supplemented with 45% haematocrit as described above at a shear rate of 500 s^− 1^. After 1, 5 and 10 min of perfusion, platelet adhesion was studied by fluorescence microscopy using a Zeiss 10fold objective compared to platelet adhesion on wt VWF biofunctionalisation. Note that no soluble VWF was present in these experiments.
2. Addressing the impact of immobilised and soluble VWF on platelet adhesion under physiological and pathological flow conditions, channels were biofunctionalised with wt VWF. Then, we perfused the channels with the washed platelet solution supplemented with 45% haematocrit as described above with or without addition of 10 μg/ml wt VWF. Perfusion was performed at distinct shear rates in the range of 1000 s^− 1^ to 10,000 s^− 1^ for 5 min. Live cell fluorescence videos were recorded with four frames per second using a Zeiss 20fold objective and analysed as described.
3. To study VWF-platelet aggregate formation, channels were biofunctionalised with wt VWF as described above. The biofunctionalised channels were perfused with native citrate-anticoagulated whole blood, additionally supplemented with indicated A-domain deleted VWF thus raising the collective VWF concentration to 50 μg/ml. The aggregation behaviour, namely the critical shear rate necessary for the formation of VWF-platelet aggregates, was then monitored as previously published [20]. Briefly, the shear rate was consecutively increased from 1000 s^− 1^ to 5000 s^− 1^ in discrete steps for 30 s each, and RICM movies were recorded with two frames per second using a specialised Zeiss 63fold antiflex objective. VWF-platelet aggregates consisting of at least 15 platelets rolling at the channel footprint were considered rolling aggregates. We determined the critical shear rate for formation of these whole blood/A-domain deleted VWF aggregates compared to those of whole blood supplemented with wt VWF. For each experiment of the aforementioned settings at least four independent experiments were performed.

### Image analysis and statistical computation

For image analysis, we used ZEN software (Zeiss AG, Jena, Germany). Calculation and quantification of the platelet SC succeeded using the open-source software ImageJ (V. 1.46r, National Institute of Health, Bethesda, Maryland, USA) analysing five randomly chosen contrast-normalised fields of view at each indicated point in time of each independent experiment. Quantification of the RICM signal intensity was also performed using ImageJ analysing contrast-normalised fields of view at each indicated point in time of each independent experiment, plotted against the time. Mean data of experiments are given with standard deviation (SD). Statistical computation was performed with SAS 9.2 (SAS Institute Inc., Cary, North Carolina, USA). Statistical significance was tested by the unpaired Student’s t-test. Significant differences of compared values are indicated by * (*P* < 0.05).

## Supplementary information

**Additional file 1.** (WMV 8497 kb)

## Data Availability

The datasets used and analysed during the current study are available from the corresponding author on reasonable request.

## Abbreviations

AVWS: acquired von Willebrand factor syndrome
BSA: bovine serum albumin
DFG: German Research Foundation
GP: glycoprotein
ICH: The International Conference on Harmonisation of Technical Requirements for Registration of Pharmaceuticals for Human Use
RICM: reflection interference contrast microscopy
SC: surface coverage
SD: standard deviation
VWD: von Willebrand disease
VWF: von Willebrand factor
wt: wildtype
*ɣ*_*crit*_: critical shear rate
